# Optimizing PRRSV Detection: The Impact of Sample Processing and Testing Strategies on Tongue Tips

**DOI:** 10.3390/pathogens14101028

**Published:** 2025-10-10

**Authors:** Igor A. D. Paploski, Mariana Kikuti, Xiaomei Yue, Claudio Marcello Melini, Albert Canturri, Stephanie Rossow, Cesar A. Corzo

**Affiliations:** Department of Veterinary Population Medicine, University of Minnesota, St. Paul, MN 55108, USA; mkikuti@umn.edu (M.K.); melin145@umn.edu (C.M.M.);

**Keywords:** PRRSV-2, epidemiology, diagnostics, alternative sample type, population-based sample, infectious diseases, swine

## Abstract

Porcine reproductive and respiratory syndrome virus (PRRSV) poses a significant challenge, costing annually approximately USD 1.2 billion to the U.S. swine industry due to production losses associated with, but not limited to, reproductive failure, abortion, and high pre-weaning mortality among piglets. PRRSV is endemic, with thirty percent of the U.S. breeding herd experiencing outbreaks annually. The shedding status of animals on a farm is typically assessed using serum or processing fluids from piglets, but tongue tips from deceased animals are emerging as a potential alternative specimen to support farm stability assessment. This study explored the impact of various processing and testing strategies on tongue tips to enhance the sensitivity and specificity of PRRSV detection in sow herds. We collected tongue tips from 20 dead piglets across seven sow farms, testing different pooling strategies (individual testing, and pools of *n* = 5 or *n* = 20) and laboratory processing methods (tongue tip fluid—TTF, versus tongue tissue homogenate—TTH). Additionally, we simulated storage and shipping conditions, comparing frozen samples to refrigerated ones tested at intervals of 1, 4, and 7 days post collection. RT-PCR testing revealed higher sensitivity and lower cycle threshold (Ct) values for TTF compared to TTH, suggesting that tongue tips are better tested as TTF rather than TTH for PRRSV detection. Pooling samples reduced diagnostic accuracy. Frozen samples had lower absolute Ct values, and Ct values increased by 0.2 Ct values each day post collection when the sample was kept refrigerated, emphasizing the importance of minimizing shipping delays. Tongue tips are a practical, easy-to-collect specimen that target potentially infected animals (dead piglets), offering valuable insights into swine herd health, but sample processing approaches significantly influence diagnostic outcomes. If tongue tips are used by veterinarians to assess viral presence on a farm, testing the TTF instead of TTH should be prioritized. Storage and shipment conditions should be considered to optimize laboratory results.

## 1. Introduction

Porcine reproductive and respiratory syndrome virus (PRRSV) is one of the most important pathogens affecting swine globally [1,2,3,4]. In the United States, the annual estimated economic losses due to PRRS are greater than USD 1.2 billion [5], with economic losses stemming from reproductive failure, abortion, premature farrowing, increased rate of stillborn piglets [6], pre-weaning mortality as extreme as 70% among piglets [7], and losses in production parameters such as post-weaning mortality, daily gain, and feed conversion [8,9].

Up to 40% of the U.S. breeding herd experiences outbreaks annually [10,11], illustrating the widespread impact of this virus in the U.S. After a PRRSV outbreak, efforts are usually made to ensure that a farm reaches stability, i.e., a lack of detectable viremia in weaning-age pigs [12,13]. This is usually achieved by “closing the herd” (preventing the entrance of naïve animals into the herd) and performing some form of immunity interventions at the herd level. The rationale is that by taking such actions, immunity on most animals will mount and circulation of PRRSV in the herd will cease. This is assessed by checking the infection status of piglets leaving the herd. The farm is considered to be “stable” when negative RT-PCR results from serum are obtained from approximately 30 piglets monthly for four months [13]. Alternative samples, including oropharyngeal and nasal swabs as well as group-level oral fluids and environmental samples [14], and more recently, tongue tips of deceased animals, have been posited as potential specimens that could be helpful in monitoring the status of a herd [15,16].

Tongue tips are a sample type that is exclusively collected from dead animals, usually piglets. When collecting tongue tips, usually the rostral third of the tongue of several dead animals on a farm is cut using a scalpel. The rationale behind tongue tip collection revolves around the animals being already dead, which makes collecting the sample a simple task. Additionally, because live animals are not handled during sample collection, the overall stress to animals is also minimized. The training required to collect tongue tips is minimal, contrasting with the training required to perform a necropsy on a dead animal, for example. Last, because tongue tips are collected from animals that are dead, and thus potentially more likely to be positive to a pathogen in case the pathogen is present on the farm, it is possible that testing tongue tips has a greater sensitivity for detecting pathogens at the herd level. Contrasted to traditionally collected oral fluids, which monitor viral circulation in live animals that are active and chewing on ropes, tongue tips allow detection of viral presence in piglets that are already dead, including stillbirths and abortions, samples that oral fluids cannot capture. Another advantage of tongue tips is that they enable sampling across multiple litters with relative ease. These potential advantages led to studies investigating the practicality of using tongue tip samples for the detection of pathogens on swine herds [14,16,17].

However, despite the ease and practicality, using tongue tips from dead animals also has disadvantages. Sample collection tends to produce a “dirtier”, more contaminated sample—tongue tips may contain many environmental contaminants and PCR inhibitors [14]. Additionally, since a variable amount of time may have elapsed since the animal’s death, tissue degradation may occur, which can potentially alter the ability to detect PRRSV via RT-PCR, since PRRSV is an RNA virus that can be degraded relatively quickly. Identifying processing techniques that improve the sensitivity of PRRSV in tongue tips as well as the conditions associated with temperature and time elapsed between collection and testing that maximize the sample sensitivity are important if this sample type is to be used on wider scale in the U.S. swine industry. Our objective was to evaluate how different sample processing and testing strategies for PRRSV detection in tongue tips affect diagnostic performance, with the goal of optimizing sensitivity and specificity in sow herds. Specifically, this study was divided to two phases in which we compared pooling strategies and processing methods to identify the most effective diagnostic approach (phase 1), and evaluated how storage temperature, shipping conditions, and time elapsed between collection and testing affect test performance (phase 2).

## 2. Materials and Methods

### 2.1. Farm Selection Criteria

This project involved a total of seven sow farms. Farms were considered eligible for the study if they had a PRRSV outbreak reported to the Morrison Swine Health Monitoring Project (MSHMP) between 2 and 5 months prior to when farms were being recruited. MSHMP is an ongoing voluntary producer-driven nationwide monitoring program for endemic swine diseases that affect the U.S. swine industry. Based at the University of Minnesota, this program collects weekly reports on the infection status of sow farms from participating swine-producing companies, veterinary practices, and regional control programs, which serves to capture the occurrence of infectious diseases in the country [10,11,18,19,20]. For this study, farm personnel were instructed to store dead piglets (including abortions) in plastic bags from the day before and the day of each visit. During each visit, tongue tips were collected from 20 dead piglets per farm. This sample size was chosen as a way to standardize how many animals would be sampled from each farm, as the number of dead animals varies across days and can not be anticipated with precision. Twenty dead piglets represented between 100% and 50% of all dead piglets these farms had on the day of the visit and the day prior. When farms had more than 20 dead piglets, animals were chosen in a manner to increase the diversity of litters they potentially came from (sampling animals of different sizes, including abortions, when those occurred).

### 2.2. Sample Collection

**Figure 1:**
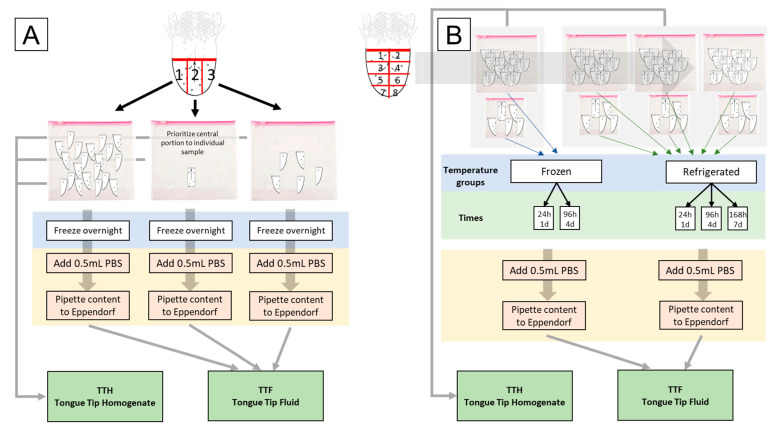
Schematic of tongue tip collection and processing prior to submission for diagnostic testing to the University of Minnesota Veterinary Diagnostic Laboratory for both phase 1 (**A**) and phase 2 (**B**) of the study.

The project was divided in two phases. In the first phase (Figure 1A), tongue tips collected from five farms were tested using different pooling strategies (pools of *n* = 20, pools of *n* = 5, or individual testing) and sample processing techniques (tongue tip fluid—TTF vs. tongue tissue homogenate—TTH). In the second phase (Figure 1B), tongue tips from two additional farms were exposed to different storage and shipping conditions (frozen vs. refrigerated) and were tested at different time points post sample collection (1, 4, and 7 days).

The tongue collection procedure differed between the two phases of the study. In phase 1 (Figure 1A), surgical rat-tooth forceps were used to expose the tongues of the animals, and approximately 4 cm of tongue was sectioned using a disposable scalpel. This section was further divided into thirds parallel to the median line on a disposable plastic surface. The median third was placed in an individual plastic container (Ziploc^®^ bag) for each animal, while the lateral thirds were pooled into containers with groups of 5 and 20 animals. Gloves and collection materials were used for a single animal and discarded after use to avoid cross-contamination between samples from different animals. In phase 2 (Figure 1B), a similar tongue tip collection procedure was followed, but the tongues were divided into eight portions using one medial cut and three subsequent lateral cuts. Each portion was randomly placed in one of four plastic containers containing pools of 5 animals and one of four containers with pools of 20 animals. Individual tongue tips were not collected during the second phase of the study.

In both phases, the collected tongues were transported from the farms to the University of Minnesota Food Centric Corridor Laboratory for further processing. During transport, samples were stored in an ice-filled, temperature-monitored cooler, with temperatures maintained at 4 °C and never exceeding 8 °C during transportation, for an average of 4 h and 15 min.

### 2.3. Sample Processing, Submission, and Testing

For phase 1 of the study, all tongue tips were frozen overnight at −18 °C (Table 1). On the following morning, the tongue tips were thawed at room temperature inside a biosafety cabinet for one hour. Due to the low volume of tissue exuded from the tongue tips, particularly those individually stored, 0.5 mL of phosphate-buffered saline (PBS pH 7.2, Gibco, ThermoFisher Scientific Inc., Waltham, MA, USA) was added using disposable Pasteur pipettes to all plastic containers containing tongues, regardless of how many tongues were stored in the container [14]. Tongues were then massaged for approximately 30 s to homogenize the PBS with the fluids exuded from the tongue tips. Using Pasteur pipettes, approximately 0.5 mL of tongue tip fluid per plastic container was then moved to 1.5 mL sterile microcentrifuge tubes (Eppendorf^®^, Hamburg, Germany). The plastic container (containing the tongue tips tissue and any leftover fluid) and the microcentrifuge tubes (containing only the tongue tips fluid) were then stored and refrigerated at 4 °C until being submitted for RT-PCR testing no more than 3 h after sample processing.

For phase 2 of the study, plastic containers containing tongue tips were either frozen at −18 °C or refrigerated at 4 °C immediately after arrival. Frozen samples from different farms were stored for either 1 or 4 days before being processed (see Table 1). Refrigerated samples from all farms were stored for 1, 4, or 7 days before being processed, representing different delays elapsed between sample collection, shipment, transportation, and arrival for diagnostic testing. Sample processing of phase 2 samples was similar to phase 1 (see yellow boxes on Figure 1).

All samples were submitted for RT-PCR testing at the University of Minnesota Veterinary Diagnostic Laboratory, where they were tested for PRRSV-1 and PRRSV-2 as part of the routine laboratory diagnosis. Samples submitted as tongue tip tissues were homogenized used using a stomacher, resulting in tongue tissue homogenate (TTH).

### 2.4. Data Analysis

The cycle threshold (Ct) value obtained via RT-PCR for each of the diagnostic tests was recorded in a database, alongside accompanying information identifying the farm from which the sample was collected, pooling strategy, sample type (TTF or TTH), temperature groups, and time elapsed since sample collection. For farms in phase 1 of the study, the sensitivity of different pooling strategies was calculated assuming that individual testing of both TTF and TTH samples was the gold standard. The Ct values of samples from TTF and TTH were compared, along with the effects of sample storage (frozen at −18 °C vs. refrigerated at 4 °C). For phase 2 of the study, a linear regression model was used to measure the expected increase in Ct values for each day elapsed post sample collection. All analyses were conducted using Stata 18 [21].

## 3. Results

A total of 140 tongue tips were collected from the seven visited farms. Since all samples tested negatively for PRRSV-1, subsequent results shown refer exclusively to PRRSV-2. In phase 1 of the study, the proportion of tongue tips that tested positive on each farm ranged from 0 to 100% when testing TTF individually and from 0 to 45% when testing TTH, also individually. The proportion of positive samples according to the sample type and pooling procedure for each farm is shown in Table 2.

Under the assumption that the individual testing of the tongue tips is the gold standard, testing TTF in pools of *n* = 5 led to an agreement with individual testing of 85%, with three cases of false negative results; while testing TTH in pools of *n* = 5 led to an agreement with individual testing of 95%, with one false positive result (Table 3). On the other hand, testing TTF in pools of *n* = 20 led to an agreement of 75%, with one false negative result; testing TTH in pools of *n* = 20 led to an agreement of 100%.

**Figure 2:**
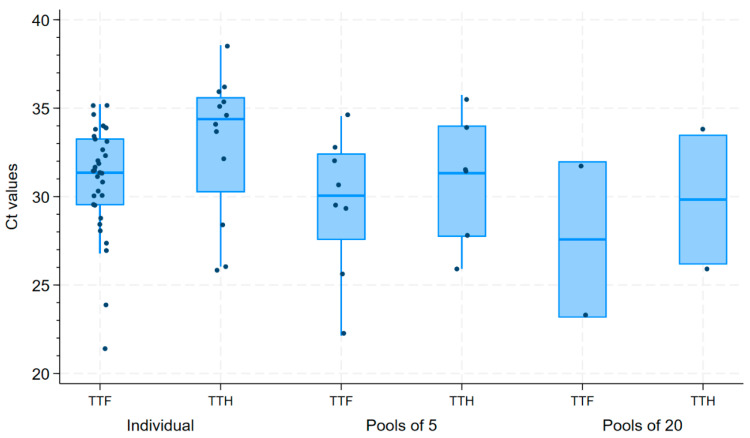
Box plot of Ct values of tongue tip fluids (TTF) and tongue tip homogenate (TTH), according to their pooling strategy.

Data from phase 1 of the study can also be contrasted on a per-sample-type basis. Under the assumption that TTF testing is the gold standard, testing of TTH had 36% sensitivity (Se), 100% specificity (Sp) and positive predictive value (PPV), and 76% negative predictive value (NPV) when samples were tested individually. When tested in pools of *n* = 5, TTH had 75% Se, 100% Sp and PPV, and 86% NPV. Both TTF and TTH performed similarly when tested in pools of *n* = 20 (data not shown). From a Ct value standpoint, TTF yielded lower Ct values than TTH, regardless of the pooling strategy. Additionally, lower Ct values were found in larger pool sizes (Figure 2).

**Figure 3:**
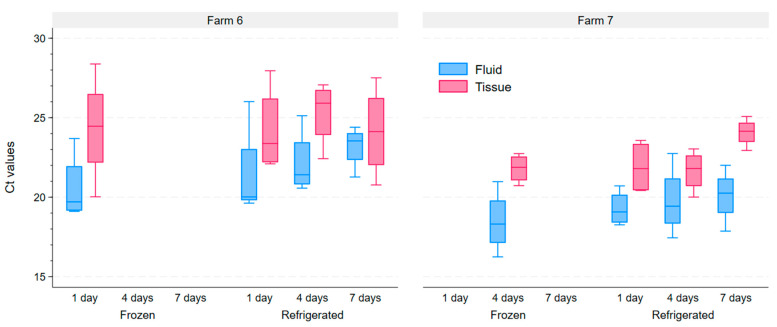
Ct values of samples tested from tongue tip fluid (blue) and tongue tip tissue homogenate (red), according to the storage condition (frozen vs. refrigerated) and time elapsed since sample collection, for both farms that participating on phase 2 of the study.

On the second phase of the study, tongue tips from pigs originating from two additional farms were used to simulate shipping conditions, allowing us to assess the effects of storage conditions (frozen versus refrigerated) and time elapsed since collection on the PRRSV-2 Ct values. The Ct values obtained from frozen samples had an absolute lower value than those obtained from refrigerated samples stored for the same period of time, although this difference was not statistically significant. We also observed a trend of increasing Ct values as the time interval between sample collection and testing increased under the refrigerated storage condition (Figure 3). Using a linear regression model, we estimate that the Ct value of samples increase on average by 0.2 units for each day elapsed between sample collection and testing (*p*-value = 0.115).

## 4. Discussion

This work examines the impact of various processing and testing strategies for PRRSV detection on tongue tips collected from dead piglets on sow farms, with the goal of optimizing diagnostic sensitivity and specificity. Our study suggests that sample processing strategies and testing conditions may affect PRRSV RT-PCR results. Specifically, testing tongue tips in pools negatively impacted PRRSV detection compared to individual tongue tip testing, as reflected in the validity measurements for PRRSV-2 diagnostics shown in Table 3. Generally, the US has seen an increase in the submission of population-based samples that are mostly being used for disease monitoring rather than diagnostic purposes [22], and it is thought that the ability that population-based samples have to test a larger number of animals may enhance the herd sensitivity [23,24]. However, our study suggests that pooling tongue tips can compromise diagnostic sensitivity and specificity (Table 3). The degree to which pooling affects these metrics likely depends on within-farm pathogen prevalence and viral load, both of which vary during an outbreak and are often unknown at the time of sampling. As the monitoring of diseases in the swine industry using population-based samples increases, it is important to highlight that the advantages of population-based samples are only likely to manifest if monitoring is carried out on sufficient animals and in a frequent enough sampling scheme, as those directly offset losses in sensitivity/specificity that a single population-based sampling scheme may have.

Overall, we observed that the Ct values on pooled tongue tips were generally lower than in individually tested ones (Figure 2), but we believe this may be an artifact from the addition of 0.5mL of PBS to all tested samples. This volume of PBS was added to all samples to allow for a sufficient volume of fluids to be tested from individually tested tongue tips, but due to small volume of sample that each individually tested tongue tip had, the addition of PBS likely led to a smaller concentration of virus per volume of tongue tip fluid. This problem was not so apparent on pooled samples, as the volume of PBS per tongue tip is lower when samples are pooled rather than tested individually.

Our study indicated that, compared to tongue tissue homogenate, tongue tip fluid testing provided lower Ct values and a higher detection rate of PRRSV-2. It is possible that the addition of cellular and extracellular components, present in higher proportions on TTH samples, are less important for PRRSV detection than saliva and blood, which are more easily found in TTF. Tongue tip fluids are the preferred sample type used when submitting tongue tips for diagnostic tests [14,15,23] or viral isolation [25], and our study further suggests that processing of the tongue involving tissue homogenization may be unnecessary, and as such, testing of TTF rather than TTH should be prioritized.

Our study highlights the importance of avoiding delays and keeping adequate storage conditions during shipping. Although there were no differences in qualitative results of the test between frozen and refrigerated samples over 7 days, the absolute Ct value obtained from refrigerated samples tended to be higher (indicating fewer genetic material particles) the longer it took between sample collection and testing (Figure 3). Other studies have investigated this and found that storing samples in higher temperatures and for longer overall lead to an increase in the Ct value obtained from them [23,26]. Delays between sampling and testing may occur at different moments. Shipping samples immediately after collection is something that veterinarians and practitioners have control over and should try to do, if possible. Delays associated to the shipment itself are generally outside of veterinarians and practitioners’ control, but risks associated with shipment can be mitigated by sending samples with adequate cooling material, as this can lead to pre-analytical errors that may be difficult to identify or control and that may affect the exact Ct value obtained. It is important to highlight that Ct values are only a proxy for genetic material present on a sample, and if precise comparisons regarding the amount of genetic material are to be made, then more precise estimates, including the utilization of standard curves for rt-PCR calibration, are needed.

Our study has several limitations. It is possible that environmental contamination of tongue tips happens. For example, piglets could chew on material containing PRRSV particles before death, leading to a positive result on tongue tips without them being infected. While this possibility cannot be ruled out, we believe its impact on practical decision-making is minimal. For practitioners, the main question is often associated with whether the pathogen is present on their farm. Tongue tip testing offers a simple, rapid, and easy-to-implement method to address that. The effect of freezing on the test results of tongue tip samples was not entirely explored, as frozen samples were not tested on all days after sample collection. However, it is well known that keeping samples frozen optimizes PRRSV RNA detection [26]. Issues associated with inadequate temperature storage are often associated with shipment delays (likely leading to thawing and storage conditions that are more similar to samples being kept refrigerated) or non-immediate mailing of samples (with on-farm storage of samples on refrigerators), both of which suggest that evaluating the effect of time on refrigerated samples may be more applicable to reality, which is why we focused on this temperature in this study.

Our study found variable PRRSV-2 prevalence between farms as assessed by tongue tips collected from dead piglets—to the extent that there were farms in phase 1 of the study in which no sample from infected piglets was collected. This minimized the contribution of that farm to the understanding of the sensitivity and negative predictive value of testing tongue tips as function of sample type or pooling strategy. That said, encountering a scenario in which all tongue tips are positive for PRRSV-2 would have precluded our ability to evaluate specificity and positive predictive value. We restricted visits to farms 2–5 months after the onset of the outbreak precisely because we wanted to maximize the variability of within-farm viral presence on the tongue tips, and we believe that introducing this source of variability made our results applicable to a broader range of real-life scenarios where PRRSV-2 prevalence is unknown and likely variable across farms over time.

Another limitation of our study is associated with the sample size, both of farms and of samples obtained within each farm. We chose to conduct the study on a total of seven farms, harvesting tongue tips from 20 dead animals on each farm. The number of seven farms was chosen in an attempt to introduce variability associated to the stage of the natural history of the outbreak each farm was in, as well as within-farm prevalence and viral load on animals while considering budget restraints. Having multiple farms allows a broader representation of scenarios, potentially allowing us to reach more generalizable conclusions than if the project was conducted on a single or fewer farms. Still, the reason for seven farms was mostly logistical, as despite Minnesota being located in the American Midwest, where most of the US swine are raised [27], identifying farms in the appropriate window of opportunity after a PRRSV outbreak that are willing to accept visits can be challenging. The choice of sampling 20 tongue tips per farm was based on previous experience from our group, in which having more than 20 dead piglets present on a farm may not always be guaranteed [14], as the number of dead piglets will depend heavily on where in the clinical course of the outbreak a farm may be and on how many animals a farm has.

## 5. Conclusions

Tongue tips are an easy-to-collect sample type that targets animals potentially more likely to be infected (dead piglets), eliminating welfare concerns during sample collection. This study provides valuable insights into how testing strategies and submission circumstances impact RT-PCR PRRSV testing results of tongue tips. While pooling samples reduces costs for producers, it also impacts diagnostic accuracy. We recommend that veterinarians discuss their specific testing objectives with pathologists, as pooling samples may still be viable depending on the question being addressed with the submission. Our study also indicates that testing tongue tip fluids yields higher detection rates with lower Ct values compared to tongue tissue homogenates. We advocate for using tongue tip fluids (rather than tongue tissue homogenate) as the primary specimen for PRRSV detection, and this information helps guiding and optimizing laboratory processing workflow of tongue tips. Additionally, our results highlight the importance of storage conditions on test performance, which is crucial when samples are shipped from farms and may face shipping delays. Minimizing the time between sample collection and testing is critical for further potential sample usage, such as sequencing or viral isolation.

## Figures and Tables

**Table 1:** Testing procedure for each farm the study. Phase 1 (farms 1–5) samples were only tested after freezing for a day, while phase 2 (farms 6 and 7) samples were tested after refrigeration of 1, 4, and 7 days and after freezing for 1 day (farm 6) or 4 days (farm 7). F represents tongue tips fluids and H represents tongue tips homogenate being tested at that point.

Phase	Farm	Storage Time		Refrigerated			Frozen		
				Pools of 5	Pools of 20		Individual	Pools of 5	Pools of 20
Phase 1	1	1d		-	-		F/H	F/H	F/H
		4d		-	-		-	-	-
		7d		-	-		-	-	-
	2	1d		-	-		F/H	F/H	F/H
		4d		-	-		-	-	-
		7d		-	-		-	-	-
	3	1d		-	-		F/H	F/H	F/H
		4d		-	-		-	-	-
		7d		-	-		-	-	-
	4	1d		-	-		F/H	F/H	F/H
		4d		-	-		-	-	-
		7d		-	-		-	-	-
	5	1d		-	-		F/H	F/H	F/H
		4d		-	-		-	-	-
		7d		-	-		-	-	-
Phase 2	6	1d		F/H	F/H		-	F/H	F/H
		4d		F/H	F/H		-	-	-
		7d		F/H	F/H		-	-	-
	7	1d		F/H	F/H		-	-	-
		4d		F/H	F/H		-	F/H	F/H
		7d		F/H	F/H		-	-	-

**Table 2:** Proportion and number of PRRSV-2-positive samples according to the sample type (tongue tip fluid or homogenate) and pooling procedure, for each of the farms on phase 1 of the study.

Farm	Proportion (n/N) of Positive Samples						
	Tongue Tip Fluid				Tongue Tip Homogenate		
	Individual	Pools of 5	Pool of 20		Individual	Pools of 5	Pool of 20
1	5% (1/20)	0% (0/4)	0% (0/1)		0% (0/20)	0% (0/4)	0% (0/1)
2	45% (9/20)	75% (3/4)	100% (1/1)		15% (3/20)	50% (2/4)	100% (1/1)
3	20% (4/20)	25% (1/4)	0% (0/1)		0% (0/20)	0% (0/4)	0% (0/1)
4	0% (0/20)	0% (0/4)	0% (0/1)		0% (0/20)	0% (0/4)	0% (0/1)
5	100% (20/20)	100% (4/4)	100% (1/1)		45% (9/20)	100% (4/4)	100% (1/1)

**Table 3:** Validity measurements of PRRSV-2 RT-PCR detection performed either on tongue tip fluids or homogenate, and contrasting different pooling strategies against individual tongue testing.

Tongue tip fluid	Individual testing		Pool of 5				Individual testing		Pool of 20		
			Positive	Negative					Positive	Negative	
		Positive	8	3				Positive	2	1	
		Negative	0	9				Negative	0	1	
		Sensitivity	72.7%	Specificity	100.0%			Sensitivity	66.7%	Specificity	100.0%
		PPV	100.0%	NPV	75.0%			PPV	100.0%	NPV	50.0%
		Agreement	85.0%					Agreement	75.0%		
Tongue tissue homogenate	Individual testing		Pool of 5				Individual testing		Pool of 20		
			Positive	Negative					Positive	Negative	
		Positive	5	0				Positive	2	0	
		Negative	1	14				Negative	0	3	
		Sensitivity	100.0%	Specificity	93.3%			Sensitivity	100.0%	Specificity	100.0%
		PPV	83.3%	NPV	100.0%			PPV	100.0%	NPV	100.0%
		Agreement	95.0%					Agreement	100.0%		

## Data Availability

The data presented in this study are available from the corresponding author upon reasonable request, subject to privacy considerations.
